# Hereditary Angioedema as a Metabolic Liver Disorder: Novel Therapeutic Options and Prospects for Cure

**DOI:** 10.3389/fimmu.2016.00547

**Published:** 2016-11-30

**Authors:** Rohan Ameratunga, Adam Bartlett, John McCall, Richard Steele, See-Tarn Woon, Constance H. Katelaris

**Affiliations:** ^1^Department of Clinical Immunology, Auckland Hospital, Auckland, New Zealand; ^2^Department of Virology and Immunology, Auckland Hospital, Auckland, New Zealand; ^3^Liver Transplantation Service, Auckland Hospital, Auckland, New Zealand; ^4^Immunology and Allergy Unit, Campbelltown Hospital and Western Sydney University, Sydney, NSW, Australia

**Keywords:** hereditary angioedema types I and II, gene therapy, CRISPR/Cas9, liver, metabolic diseases

## Abstract

Hereditary angioedema (HAE) is a rare autosomal dominant disorder caused by mutations of the SERPING1 or the Factor 12 genes. It is potentially fatal, particularly if not identified at an early stage. Apart from androgens, which are contraindicated in children and in pregnant women, a range of effective, albeit very expensive treatments have recently become available for HAE patients. The cost of these new treatments is beyond the reach of most developing countries. At this time, there is no cure for the disorder. In spite of mutations of the SERPING1 gene, autoimmunity and infections are not prominent features of the condition. Here, we present the argument that HAE should be viewed primarily as a metabolic liver disorder. This conceptual paradigm shift will stimulate basic research and may facilitate new therapeutic approaches to HAE outlined in this paper. We suggest several novel potential treatment options for HAE from the perspectives of clinical immunology, molecular biology, and liver transplantation. Many of these offer the prospect of curing the disorder. The effectiveness of these options is rapidly improving in many cases, and their risks are decreasing. Given the very high costs of treating HAE, some of these curative options may become feasible in the next decade.

## Introduction

Hereditary angioedema (HAE) is rare disorder caused by mutations of the SERPING1 or F12 genes. It was first described by Quincke in 1882 (1) and subsequently by Osler (2). Affected patients experience recurrent abdominal pain or swelling, which can be fatal if it involves the larynx. Current estimates suggest a prevalence of about 1:70,000 in the general population (3). There is no obvious ethnic variation in the disorder. Typically, symptoms begin in late childhood and become worse after puberty. Therefore, the disorder has a much higher morbidity and mortality in adults compared with children.

Hereditary angioedema is an autosomal dominant disorder, where heterozygotes are symptomatic. There are examples of variable penetrance and expressivity of the gene, where some affected family members may not manifest any symptoms. As discussed below, there are recent data suggesting sequence variations in genes involved in the production or catabolism of bradykinin might also influence the severity of the disorder. This phenotypic variability can contribute to the delay in diagnosis of the condition, which is typically about 8 years (4, 5). There is a high mortality rate in HAE patients prior to diagnosis (6).

Angioedema attacks can be triggered by trauma, stress, and hormonal changes associated with menstruation or pregnancy in affected women. There is evidence for systemic effects of local activation of the contact phase (7). Estrogens and angiotensin-converting enzyme (ACE) inhibitors can also trigger attacks, the latter by interfering with metabolism of bradykinin. ACE is the dominant enzyme responsible for degrading bradykinin.

It is clear that androgen treatment can reduce the frequency and severity of the attacks (8). However, androgens are contraindicated in children and pregnant women and can cause troublesome virilizing adverse effects in women (8). In the last few years, there have been remarkable advances in management of the disorder (9). Use of purified or recombinant C1 inhibitor (C1 INH) is effective in treating severe attacks, especially if administered early. Other drugs, such as bradykinin receptor, antagonists, and kallikrein inhibitors, have also proven to be effective in treating acute attacks (10). Unfortunately, the high cost of these newer medications places them beyond the reach of most developing countries (Table 1). In spite of these advances, a cure for this potentially fatal disorder has been elusive.

**Table 1:** **Comparison of currently available treatments for HAE types 1 and 2**.

Current treatment	Cost/year	Benefits	Risks	Comment
Fibrinolytic inhibitors	Cheap	Cheap	Risk of thrombosis in pregnancy. Partially effective	Can be used in children who develop HAE symptoms early
Androgens	Cheap	Cheap	Contraindicated in pregnancy, children. Hepatic adenomas, lipids	Virilizing adverse effects in females
C1 inhibitor prophylaxis	High	Effective	Risk of pathogen transmission (not with recombinant C1 INH)	Unaffordable for most countries
Bradykinin receptor antagonists	High	Effective for acute attacks	Pain at local site	Unaffordable for most countries
Kallekrein inhibitors	High	Potential for oral treatment	Systemic reactions for parenteral preparations	Unaffordable for most countries

*The costs for these medicines vary in different countries*.

Recurrent infections or autoimmunity are not a feature of the disorder. Yet, in the most recent IUIS/WHO classification of primary immunodeficiency disorders, HAE is listed as an immunodeficiency disorder because the mutated gene functions as an inhibitor of the complement cascade (11). The dominant biochemical problem is impaired function of the contact phase leading to unregulated production of bradykinin (12), which in turn causes the angioedema. Although useful for making the diagnosis, the complement cascade is mostly a bystander in swelling attacks. Dysfunction of the complement system does not lead to immediate symptoms or major complications in HAE. Sequence variations in SERPING1 have been linked to age-related macular degeneration (13).

**Figure 1:**
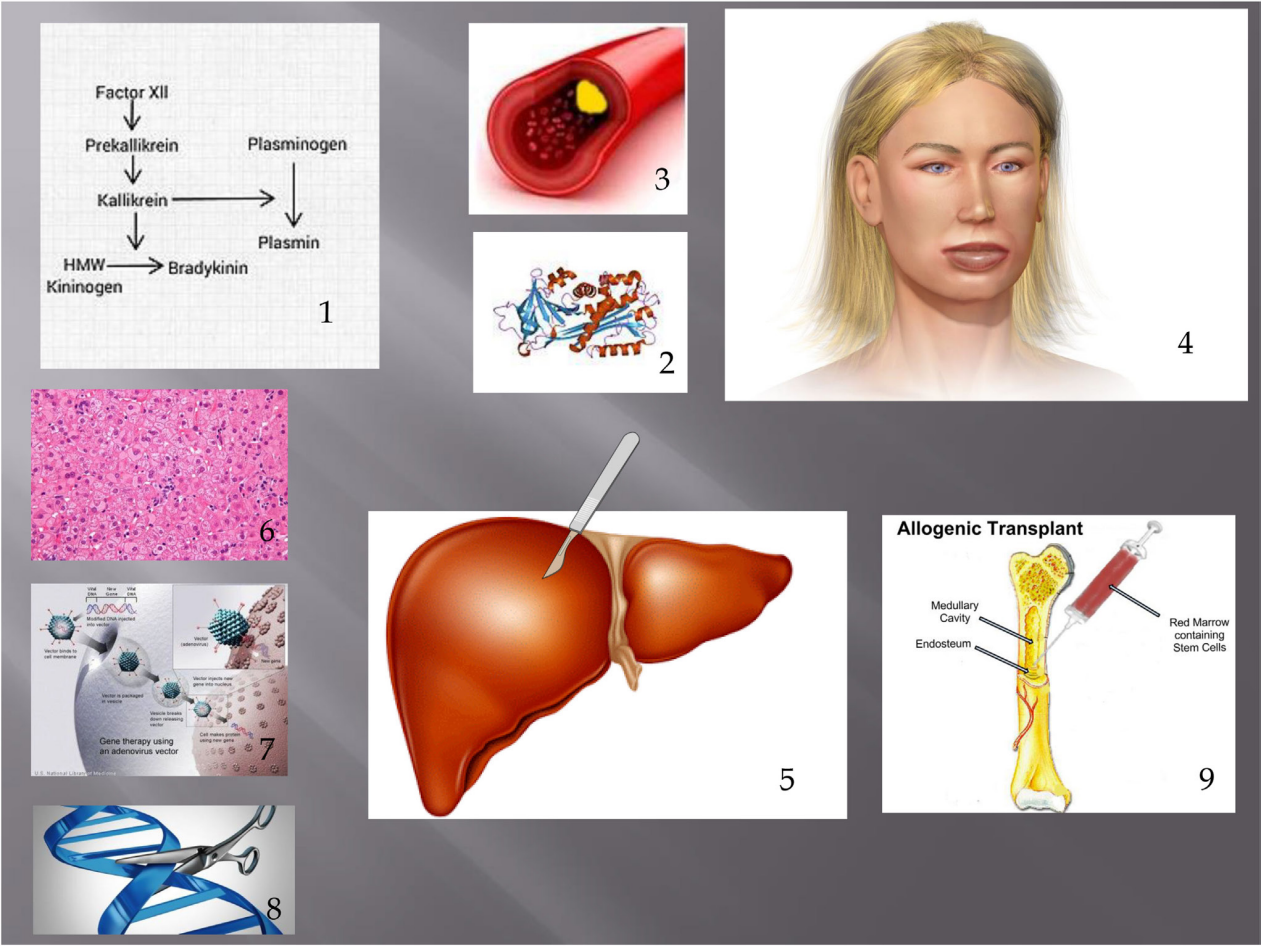
**Showing pathogenesis of HAE and treatments discussed in this article**. **(1)** Contact phase. In HAE, an excess of bradykinin is present as a result of impaired C1 INH function. **(2)** This leads to extravasation of fluid from blood vessels **(3)** leading to angioedema. **(4)** Possible treatments discussed include liver transplantation (including APOLT), **(5)** hepatocyte transplantation, **(6)** liver-based gene therapy, **(7)** and genome editing. **(8)** Bone marrow transplantation has also led to a cure of HAE. **(9)** Figure constructed with clipart from Microsoft PowerPoint.

Here, we present the argument that HAE should be conceptualized as a metabolic liver disorder. This is likely to assist with research, which we believe may result in a cure for our patients within our lifetimes. While we discuss these technologies/treatments as possible alternatives to current treatment (Figure 1), it is essential that the risks of these approaches (Table 2; Figure 1) are carefully considered and technical issues are resolved before human trials are undertaken. The most important principle is primum non-nocere.

**Table 2:** **Potentially curative options for patients with types 1 and 2 HAE**.

Potential curative treatments	Cost/year	Benefits	Risks	Comment
Liver transplantation	High initial cost and moderate ongoing costs	Potentially curative	Peri-operative risks and long-term immunosuppression	Dependent on transplanted liver
APOLT	High initial cost and moderate ongoing costs	Potentially curative	Peri-operative risks and long-term immunosuppression	Native liver preserved so immunosuppression could be withdrawn if alternative therapies became available
Hepatocyte transplantation	High	Minimally invasive	May require immunosuppression. May fail over time	Experimental
AV			Unacceptable risks	
AAV	High	Allowing vector entry into hepatocytes. Could be used for gene editing or to deliver wild-type gene	Approved for FIX deficiency	May need ongoing immunosuppression
Stealth viruses	Very high	No need for immunosuppression. Could be used to deliver wild-type gene	Endogenous hence less likely to be antigenic	Customized viral vector needed. Probably not practical for routine use
CRISPR Cas9	High	Potential for correcting mutation	Serious risk of off-target effects	Unlikely to a practical option in the near future

*The cost of liver transplantation varies in different countries*.

*APOLT, auxiliary partial orthotopic liver transplantation; AV, adenovirus; AAV, adeno-associated virus*.

## Understanding Transinhibition as a Critical Component of the Molecular Basis of Type 1 HAE

A better understanding of the molecular pathology of HAE may result in the availability of more effective therapies for the disorder. It is now apparent that HAE is a genetically complex disorder. It belongs to the serpinopathies, as exemplified by alpha1 antritrypsin deficiency. In addition to mutations of the SERPING1 gene, sequence variations in other genes such as F12, peptidases, and bradykinin receptors may influence the phenotypic severity of the disorder (14).

For the purposes of this review, we shall be focusing on type 1 and type 2 HAE. HAE with normal C 1 INH (previously known as type 3 HAE) appears to have a different pathogenesis. Some of these patients have causative mutations of the F12 gene, while the pathogenesis of the disorder is unknown in other HAE patients (14). While some of the therapeutic options suggested here may be useful for F12 gene mutations also, less is known about this condition than types 1 and 2 HAE. In this perspective, we shall therefore be focusing on types 1 and 2 HAE.

Potential curative options are critically linked to the molecular biology of the disorder, particularly transinhibition. Type 1 and type 2 HAE are caused by mutations of the SERPING1 gene. Over 400 different mutations have been described in the condition (15). Given the autosomal dominant inheritance, affected patients have one mutated and one wild-type allele. In type 1 HAE, C1 INH levels are very low or absent in the blood. In type 2 HAE, levels of C1 INH are normal or elevated. Type 2 HAE is thought to be a dominant-negative disorder, caused by a circulating dysfunctional protein, which competitively inhibits wild-type C1 INH. Some patients with type 2 HAE also produce aggregates of mutant C1 INH multimers, which have the potential to directly activate the contact phase, leading to further bradykinin production (16).

Based on the molecular pathology, patients with type 2 HAE might be expected to have missense mutations of the SERPING1 gene allowing expression of a dysfunctional protein causing a dominant-negative effect, and patients with type 1 HAE might be expected to have severe mutations such as nonsense mutations or complex rearrangements leading to haploinsufficiency. This is not invariable (17). In spite of one wild-type allele, patients with type 1 HAE may have low circulating C1 INH. In type 2 HAE patients, the functional C1-INH plasma levels are between 5 and 30% of the normal range, as opposed to the 50% as may be expected in heterozygotes (18). While there may be some evidence for excess consumption (17), there is evidence for transinhibition of the wild-type protein by the mutant allele in type 1 HAE (19). Gene expression profiling has shown that mutant as well as wild-type transcripts were reduced in HAE patients (20).

An important publication over 20 years ago addressed this observation in fibroblasts from HAE patients (19). The authors reported a patient with a deletion of exon 7 that allowed comparison of wild-type vs. mutant allele expression. It appears that both transcription and translation of the wild-type allele were impaired. A similar phenomenon was seen in HAE mice generated by gene targeting (21). There has not been further exploration of this important observation. The precise mechanism of how transinhibition occurs in HAE has not been elucidated. The transinhibition of SERPING1 does not appear to influence the expression or production of other hepatic proteins.

It is apparent that wild-type C1 INH levels increase in HAE patients who have been treated with androgens. Androgens also appear to increase the levels of Aminopeptidase P, which in turn accelerates catabolism of bradykinin (22). The molecular mechanism for these effects has not been elucidated *in vitro*. Understanding the molecular basis of these actions may offer insights into the pathogenesis of the disorder and lead to new and more effective treatments without the troublesome adverse effects of androgen therapy. It is also apparent that some cytokines such as IL6 can result in substantial upregulation of hepatic C1 INH production (23). It may be possible to design a biosimilar, which upregulates C1 INH production, without the deleterious adverse effects of IL6 on B cells and other target cells.

## HAE as a Metabolic Liver Disorder: Options for Liver or Hepatocyte Transplantation

Given that C1 INH appears to be produced predominantly in the liver, HAE could be considered a metabolic liver disorder (Table 2; Figure 1). Liver transplantation is a proven effective treatment for other potentially fatal metabolic liver disorders (24, 25). However, the procedure carries significant surgical risks and transplanted patients require long-term immunosuppression to prevent graft rejection. These drugs have a variety of adverse effects including predisposition to infections, impaired renal function, and an increased risk of some malignancies. The shortage of donor organs also limits the availability of liver transplantation as a therapeutic option (26).

Even though liver transplantation would be expected to be curative for HAE, we have not been able to identify any reports of patients who have undergone liver transplantation either for HAE or for a coincidental indication. However, we have identified an instance where a patient developed type 1 HAE after receiving a liver from a donor with HAE (27). This observation is strongly indicative that HAE could be cured by liver transplantation.

It may be argued that patients with HAE have other effective treatments and therefore should not be considered for liver transplantation. However, the results of liver transplantation are continuing to improve, and alternative therapies are expensive and have limited availability. Use of prophylactic C1 INH can cost over US $200,000 per year. The costs of liver transplantation varies widely and certainly after the upfront cost of the surgery, and the annual cost of prophylactic C1 INH would exceed that of post liver transplantation care (Table 2). If a patient with HAE has a standard indication for liver transplantation, they should receive priority as the liver transplant may cure both disorders.

Auxiliary partial orthotopic liver transplantation (APOLT) could potentially be used in patients with type 1 HAE (28). This procedure carries a lower post-procedure risk than liver replacement because if the graft fails, the native liver is retained and is able to provide other essential functions. While transinhibition may be taking place in type 1 HAE patients’ livers, the graft may produce sufficient C1 INH to control the contact phase. This may be less effective in patients with type 2 HAE, where large amounts of dysfunctional C1 INH are produced and could outcompete the wild-type C1 INH produced by the graft. However, even a small change in the ratio of wild-type to mutant C1 INH levels may ameliorate the disorder in type 2 HAE patients as seen with androgen treatment. As discussed below, C1 INH could be given by regular subcutaneous infusion and functional C1 INH levels could be measured to determine the threshold at which symptoms are ameliorated. This may allow better selection of patients for some of the interventions proposed here.

Hepatocyte transplantation is another potential approach. Here, individual hepatocytes are isolated from the donor liver (29) and then infused into the recipients’ livers *via* either the portal vein or the umbilical vein or peritoneum in neonates. Engrafted hepatocytes would be expected to produce proteins including C1 INH. Again, this option may be more effective in patients with type 1 HAE than those with type 2 HAE. Although hepatocyte transplantation has been undertaken for a variety of metabolic liver diseases, there are still significant barriers to long-term engraftment and hepatocyte transplantation remains an experimental procedure (29). Hepatocyte transplantation also requires immunosuppression, and some hepatocytes may dedifferentiate and cease to produce proteins, leading to eventual therapeutic failure (29).

Recent advances in tissue engineering such as use of decellularized organ matrices and 3D printing may pave the way to new alternatives in treating liver diseases (30). Technical advances would be needed before this procedure becomes a routine treatment for HAE. The risks of long-term immunosuppression need to be balanced against availability of effective drugs to treat HAE.

## Liver-Based Gene Therapies

Two decades ago, there was considerable interest in liver gene therapy (31). In most trials, a vector, usually an Adenovirus, carrying a wild-type copy of the defective gene was introduced into hepatocytes. The wild-type protein was expressed in the cells to rectify the defective gene. Non-integrating vectors with the wild-type gene insert do not integrate into the host genome; hence, permanent and controllable gene expression was problematic.

The vector manipulation was able to be undertaken *ex vivo* or *in vivo*. An *ex vivo* gene therapy protocol was trialed in patients with type 2 familial hypercholesterolemia (32). Hepatocytes were isolated from the resected left lateral lobe, cultured, and genetically modified. The transgenic hepatocytes were then transplanted back to the patients. The treatment led to modest decrease in the LDL cholesterol. This strategy was hampered by technical difficulties with the genetic manipulation and the low number of transplanted hepatocytes which successfully integrated *in vivo* (33).

In other liver-based gene therapies, the recombinant virus was infused into the patient after being prepared *in vitro*. The virus infected the target cells and subsequently the host cell machinery produced the therapeutic protein. Enthusiasm for this strategy was seriously dampened after the death of 18-year-old Jesse Gelsinger, a patient with mild ornithine transcarbamoylase (OTC) deficiency. Jesse was a mosaic for OTC and had a mild disorder. He was enrolled in an Adenovirus gene therapy trial at the University of Pennsylvania. Within 3 days of gene therapy, he had a catastrophic immune response to the adenoviral vector and died of multi organ failure (34). A subsequent FDA investigation uncovered protocol issues as well as potential conflicts of interest (35). A lawsuit was settled out of court. The chief investigator has courageously shared his insights into the tragedy for the benefit of future participants and investigators in such trials (36).

Subsequently, trials of adeno-associated viral (AAV) vectors have proved safer and more effective (37). This group of parvoviruses is commonly found in humans and is non-pathogenic. The AAV vector does not elicit an intense antiviral immune response. It is therefore considerably less immunogenic than the previous Adenoviral vectors. Patients with hemophilia B have been successfully treated with this approach with long-term improvement in bleeding diathesis. A 1–5% increase in FIX production has been sufficient to ameliorate bleeding. A number of AAV/hemophilia B trials are currently in progress (38, 39). In these studies, F12 cDNA (1.4 kbp) was incorporated, packaged into an AAV8 vector, and infused into patients.

Similarly, C1-INH cDNA (1.5 kbp) could potentially be delivered into patients *via* an AAV8 vector. It is possible that even an increase of 5% wild-type C1 INH expression may modify the angioedema tendency of type 1 HAE patients, as has been achieved in FIX deficiency. This is similar to quantities that would be expected with androgen therapy. Recent studies have shown that maintaining functional C1 INH levels above 40% prevents angioedema attacks (23). It may be possible to achieve this with liver-based gene therapy. Perhaps, patients could be selected based on their baseline functional C1 INH levels. Patients who are most likely to benefit may be those with functional C1 INH levels of 20–30%. As suggested above, these patients could be given a trial of subcutaneous C1 INH to confirm that levels >40% result in reliable suppression of angioedema attacks (23).

Although the AAV vector is considerably less immunogenic than the previous Adenoviral vectors, it can still provoke an immune response leading to elimination of transfected cells (40). Furthermore, previous exposure to AAV can result in formation of neutralizing antibodies, which may impair the efficacy of the AAV-mediated gene transfer. A late T cell response can also lead to elimination of transfected hepatocytes and provoke a transient hepatitis. Some protocols use immunosuppression at the time of vector infusion to impede the immune response. Immunosuppressive drugs carry severe long-term risks including permanent immunosuppression and will need to be used with caution.

Efforts are underway to further reduce the immunogenicity of the vector by modifying or deleting other viral proteins (41). An alternative approach is the isolation and modification of endogenous AAVs, which have already infected patients and use these as customized vectors. These viruses are likely to be seen as self and are unlikely to provoke a severe T or B cell response. These “stealth” virus vectors may prove safe and efficacious but will involve considerable expense as each patient would require creation of their own customized vector. Immunosuppression may not be needed with this approach.

There is considerable interest in other new viral and non-viral approaches for gene therapy in liver disease (31). Most human studies are at a proof-of-concept stage. Again, the use of this technology will need to be balanced with the effective drugs currently available for HAE. AAV gene therapy for Hemophilia B was recently approved by the FDA, underscoring the potential of these approaches.

Animal studies will be needed to ensure that overexpression of the C1 INH does not cause problems with the contact, fibrinolytic, or complement systems. This would seem unlikely given that many patients are now on long-term prophylactic C1 INH treatment and do not suffer adverse effects in other systems. Furthermore, overexpression of transfected genes has not been seen any liver-based gene therapy trial to date and is unlikely to be a clinical problem.

## *In Vivo* Gene Editing Including the Crispr Cas9 System

The ultimate goal of gene therapy is to enable long-term expression of the corrected gene under the control of endogenous regulatory elements to cure the disorder. The newest frontier of gene therapy is *in vivo* genome editing. This technique allows researchers to carry out site specific modification of the host genome. Numerous papers have reported encouraging results from cellular and murine studies (42).

The concept of clustered, regularly interspaced, short palindromic repeat (CRISPR)-mediated genome editing originates from the bacterial adaptive defense system against bacteriophage attack. CRISPRs in bacterial genomes consist of sequences of viral origin (43). In this system, bacteriophage sequences have been incorporated into bacterial genomes. When attacked by bacteriophages, bacteria are able to transcribe these sequences which then hybridize to the phage nucleic acids. The Cas series of enzymes are then able to degrade the phage DNA.

This system has been adapted for modifying genomes of eukaryotic cells (43, 44). With the use of guide RNAs, mammalian sequences can be targeted and edited. By editing target genes, mutations can be corrected *in vivo*. Hereditary tyrosinemia caused by fah mutations has been corrected in mice with a vector bearing the wild-type gene and the Cas9 enzyme (45). Even a slight change in ratio of wild type: mutant transcripts may alter the phenotype leading to a milder version of HAE. In hepatocytes where the mutation has been corrected, transinhibition will no longer apply, which may lead to a disproportionate increase in wild-type C1 INH transcripts and protein. This is supported by an interesting observation, where HAE type 1 was corrected by bone marrow transplantation. The amount of C1 INH produced by the transplanted macrophages is likely to be minor compared with that produced in the liver. This observation supports the notion that even a small increase in wild-type C1 INH by any method may alter the phenotype of the patient (46).

Other gene-editing methods have also been trialed. A proof-of-concept study was recently published where the CCR5 gene in CD4 cells from HIV patients was modified with a Zn finger nuclease to prevent HIV infection (47). CCR5 is a co-receptor for HIV entry into CD4 cells, and mutations can confer resistance to HIV (48). In this uncontrolled study, there was a rapid increase in CD4 cells and a corresponding reduction in HIV viral load. Zn finger nucleases offer an alternative method to CRISPR Cas9 for gene editing.

Perhaps, a combination of an AAV carrier with a plasmid carrying the CRISPR Cas9 or Zn finger nuclease may prove effective in the future for some HAE patients. Promising developments have been reported in the animal literature. Zinc finger nucleases, delivered as part of the AAV viral package *via* the tail vein, have been shown to deliver target genes to the liver leading to gene correction in a hemophilia B mouse model.

A screening study identified Cas9 from *Staphylococcus aureus* (SaCas9), which exhibits similar DNA editing efficiency to the present system with *S. pyogenes* Cas9 (SpCas9) (49). SaCas9 is 25% smaller than SpCas9 and could be packaged more efficiently into the AAV viral capsule. Ran et al. used the SaCas9/AVV to target cholesterol regulatory gene Pcsk9 in the mouse liver (49). They reported a reduction in serum Pcsk9 and cholesterol levels.

Although gene editing shows great promise for treating patients with genetic disorders (50), serious technical issues may limit the safe, widespread application of the technology. In the CRISPR Cas9 system, the guide RNA is critical in preventing off-target effects. In this system, it may be difficult to detect off-target effects in all modified cells. Even a single aberrantly gene-edited cell could clonally expand and give rise to malignancy, akin to previous gene therapy studies in SCIDX1 patients (51). A recent study of gene editing of non-viable human embryos showed a high incidence of off-target effects (52).

Furthermore, there may be a risk of immunological reactions against the Cas9 nuclease. There may be a risk of transient or chronic hepatitis. Currently, this technology can only be applied to small mutations. Complex rearrangements including large deletions and inversions cannot be corrected with the CRISPR Cas9 system at this time. Up to 20% of patients with type 1 and type 2 HAE have complex mutations that would not be amenable to current gene-editing techniques.

Other approaches may, however, be useful when there are complex gene rearrangements, such as those seen in the 20% of HAE patients with complex mutations. Chromosomal inversion in factor VIII (F8) causes almost half of all severe hemophilia A. Park et al. reported using transcription activator-like effector nucleases (TALEN) to revert an inverted 140 kbp mutation to its original orientation in human-induced pluripotent stem cells (iPSCs). The approach also restores FVIII mRNA production (53).

The efficiency of gene editing may also be a problem for patients with HAE. In the case of hereditary tyrosinemia and HIV, the gene correction frequency was low, but the gene-edited cells had a proliferative/survival advantage. This may not be the case in HAE. However, as shown above, a small number of gene-corrected cells may be sufficient to ameliorate the angioedema phenotype.

The serious safety issues of *in vivo* gene editing will need to be addressed. Strategies for mutation correction with high efficiency but zero risk of off-target effects are unlikely to be established in the immediate future. This technology will have to overcome major technical and safety barriers before it could be used routinely for correcting genetic defects in human diseases. It would seem most prudent to initially trial these technologies in otherwise lethal diseases, for which there is no current treatment, rather than HAE.

## HAE Prevention

In addition to the technologies discussed above, prevention of HAE is possible with prenatal diagnosis and preimplantation genetic diagnosis (PGD) (54). The genetic diagnosis must be established before considering these options (55–57). Patients must undergo intensive counseling before these options are considered. The possibility of having mildly affected progeny and the stress associated with *in vitro* fertilization must be considered prior to PGD. Female patients with HAE may be vulnerable to angioedema attacks during hormonal stimulation in the egg harvesting phase of IVF and will need prophylactic C1 INH. Given that 25% of patients are the result of new mutations, this strategy will not prevent all cases of HAE. Furthermore, in some patients, the mutation cannot be identified, and this will also limit the numbers of patients who could be offered prenatal diagnosis and/or PGD.

## Conclusion

In the future, safe, cheap, and effective oral treatments may become available for patients with HAE. Apart from oral androgens, with their well-recognized adverse effects, most other prophylactic treatments are prohibitively expensive for most countries (Table 1). Regarding HAE as a metabolic liver disorder may facilitate new therapeutic approaches to prevent or cure the disorder (Table 2; Figure 1). Elucidating the basis of transinhibition will be the key to understanding the molecular pathology of HAE. There may be a range of curative options available to physicians in the future. These options are likely to depend on the nature of the mutation in individual HAE patients. Clearly, much more research is needed before these technologies can be safely offered clinically. Given the lessons of the past, we suggest these options are deployed with great caution.

## Author Contributions

RA conceptualized the review. AB and JM contributed to the liver transplantation literature. S-TW contributed to the molecular aspects of the review. CK and RS provided information on clinical aspects of the perspective.

## Conflict of Interest Statement

The authors declare that the research was conducted in the absence of any commercial or financial relationships that could be construed as a potential conflict of interest.
